# Analysing 454 amplicon resequencing experiments using the modular and database oriented Variant Identification Pipeline

**DOI:** 10.1186/1471-2105-11-269

**Published:** 2010-05-20

**Authors:** Joachim M De Schrijver, Kim De Leeneer, Steve Lefever, Nick Sabbe, Filip Pattyn, Filip Van Nieuwerburgh, Paul Coucke, Dieter Deforce, Jo Vandesompele, Sofie Bekaert, Jan Hellemans, Wim Van Criekinge

**Affiliations:** 1 Laboratory for Bioinformatics and Computational Genomics, Department of Molecular Biotechnology, Ghent University, 9000 Ghent, Belgium; 2 Center for Medical Genetics, Ghent University Hospital, 9000 Ghent, Belgium; 3 Department of Applied Mathematics, Biometrics and Process Control, Ghent University, 9000 Ghent, Belgium; 4 Laboratory for Pharmaceutical Biotechnology, Faculty of Pharmaceutical Sciences, Ghent University, 9000 Ghent, Belgium; 5 on behalf of the NXTGNT collaborators, Ghent University, 9000 Ghent, Belgium

## Abstract

**Background:**

Next-generation amplicon sequencing enables high-throughput genetic diagnostics, sequencing multiple genes in several patients together in one sequencing run. Currently, no open-source out-of-the-box software solution exists that reliably reports detected genetic variations and that can be used to improve future sequencing effectiveness by analyzing the PCR reactions.

**Results:**

We developed an integrated database oriented software pipeline for analysis of 454/Roche GS-FLX amplicon resequencing experiments using Perl and a relational database. The pipeline enables variation detection, variation detection validation, and advanced data analysis, which provides information that can be used to optimize PCR efficiency using traditional means. The modular approach enables customization of the pipeline where needed and allows researchers to adopt their analysis pipeline to their experiments. Clear documentation and training data is available to test and validate the pipeline prior to using it on real sequencing data.

**Conclusions:**

We designed an open-source database oriented pipeline that enables advanced analysis of 454/Roche GS-FLX amplicon resequencing experiments using SQL-statements. This modular database approach allows easy coupling with other pipeline modules such as variant interpretation or a LIMS system. There is also a set of standard reporting scripts available.

## Background

Recent DNA sequencing technology, the so-called next-generation sequencing (NGS) technology, enables researchers to read a number of DNA sequences that is several orders of magnitudes bigger and at a cost that is several orders of magnitude smaller than the previous generation DNA sequencing technologies. The cost of determining the human genome was estimated at $2.7 billion for the IHGSC genome and at $300 million for the Celera genome. Recently several human genomes were sequenced in about 1.5 months at a cost that is around $1.5 million [1,2].

Large-scale parallel pyrosequencing from 454/Roche generates hundreds of thousands sequenced DNA reads within a matter of hours [3]. The latest version of the sequencing technology (Titanium) enables a throughput of 0.4-0.6 gigabases per 10 h run [4]. The amount of data to be analyzed keeps growing at an increasing speed. Other NGS platforms such as Illumina's Genome Analyzer (San Diego, CA, USA), Applied Biosystems' SOLiD (Foster City, CA, USA) and Helicos' HeliScope (Cambridge, MA, USA) generate more than 10 gigabases in a single multi-day run. It is estimated that by 2012 genome centres around the world will generate more data per year than the expected 15 petabytes per year that is produced by CERN's Large Hadron Collider [5].

Sequencing researchers agree that data-analysis of NGS projects is the biggest challenge to make the technology accessible for biologists around the world. The cost of sequencing might go as low as $1,000 per human genome, but it will still be a lost investment if the generated data cannot be adequately analyzed. Especially sequencing platforms with a very high throughput of read lengths as short as 35-40 nucleotides face challenges for genome assembly and annotation [6].

Nevertheless, NGS is expected to have an enormous impact on diagnostics and SNP discovery [7], provided there are tools available that make variant detection and interpretation of the sequenced data straightforward and automated. A good step towards standardization and data uniformity, especially in the short-read field, was taken with the development of the SAM/BAM format [8]. This format is currently supported by BWA [9] and Bowtie [10], but is not supported by long-read (typically 454 GS-FLX data) mappers such as BLAT [11]. However, with the recent release of BWA-SW [12] one might expect a more broad transition of long-read NGS towards SAM/BAM pipelines.

There are already some tools available to analyze 454 GS-FLX experiments, but mostly with a limited analysis spectrum. Most of the present tools are designed to do a specific task, and rarely offer broad spectrum analysis. The few integrated analysis tools offering broad spectrum analysis available are commercial packages (such as CLCbio Genomics Workbench, Genomatix or NextGENe).

Mosaik [13] for example is one of the few broader analysis packages available and has cross-platform features which makes it indeed easy to work with. Unfortunately it lacks some additional analysing possibilities besides mapping and assembly that are useful in amplicon resequencing based diagnostics (for example coverage per amplicon and per patient or calculation of primer dimer frequencies).

Roche's Amplicon Variant Analyzer (AVA) software is specifically designed for analysis of amplicon resequencing experiments and is user-friendly but has some limitations. Advanced coverage analysis is of paramount importance in a diagnostic setting but is lacking in AVA. Data storage is also an issue using AVA as data is not stored in a structured way (i.e. flat files instead of storage in a database).

In this paper we describe an open-source database oriented pipeline that is capable of analyzing a single GS-FLX amplicon resequencing run automatically from receiving the raw data to generating custom (variation) reports within one day. Using the database approach, the pipeline is capable of analyzing a set of runs together in a meta-analysis. While sequence variations can be assessed using this pipeline, it also allows researchers in diagnostic labs to extract additional information (e.g. frequency of primer dimers). This data can then be used to optimize the amplicon PCR reactions and general sequencing settings to improve future sequencing runs. The pipeline, a manual, a testing dataset, and example reports are available on the web at http://athos.ugent.be/VIP_pipeline.

## Implementation

**Figure 1:**
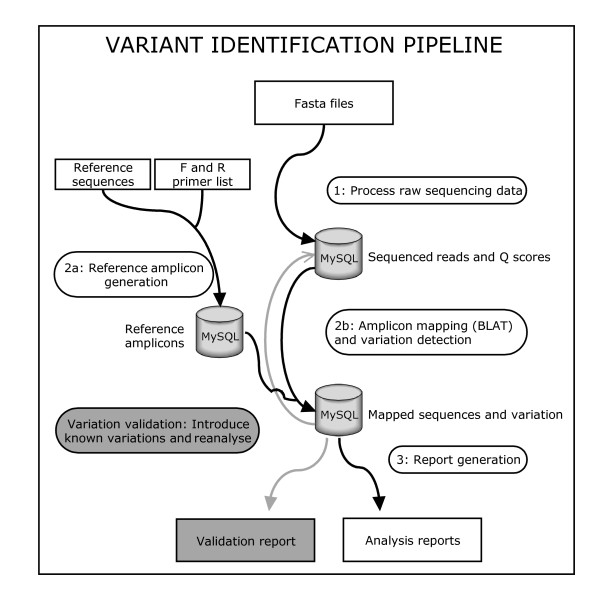
**Overview of the VIP pipeline**. Overview of the Variant Identification Pipeline (black arrows and white text-boxes) and the VIP Validator (grey arrows and grey text-boxes). The analysis pipeline consists of 4 modules. 1) Raw sequences are extracted from the FASTA files generated by the GS-FLX sequencer and processed into sequenced amplicons and additional information. 2a) Reference amplicons are generated using a list of reference sequences and the list of primers. 2b) Mapping is carried out with BLAT using the reference amplicons and the sequenced amplicons. Variations are detected and stored in the database. 3) The requested reports are generated. The VIP Validator introduces additional variation in the sequence reads and reanalyses those sequences to validate the pipeline for that specific variation.

The Variant Identification Pipeline (VIP) is developed in a modular way so that each module can run independently from another. Each module uses data stored in a relational database (MySQL) to do its task and stores its result again in the database (Figure 1). The pipeline is completely written in Perl and the DBI and DBD::mysql packages are used for database interactions.

### Processing the raw sequence data

The pipeline uses both the FASTA (sequenced reads) and FASTQ (positional quality scores of the sequenced reads) files generated by the 454/Roche GS-FLX sequencer as input and processes the files in a way so that a DNA sequence is linked to a sequence of quality scores. In a first step the DNA sequence is trimmed using a variety of algorithms. The multiplex identifier or MID (a short bar-code sequence used to label samples/patients when multiplexing), the linker sequence (used to link the amplicon primer together with the MID sequence), and the trailing sequence (residual reverse complements of the MID/linker at the end of a sequence) are trimmed. The different algorithms allow perfect tag sequences to be trimmed but also tag sequences with sequencing errors.

Trimming the sequences results in more efficient mapping in later stages of the pipeline as artificial nucleotide strings are removed from the sequence before they can interfere with the mapping process. The combined length of the MID and linker is often >20 bp and can cause problems in mapping, especially when amplicons are already rather short.

### Generating the reference amplicons

Both a reference sequence and a list with forward and reverse primers (in FASTA format) are fed to the amplicon generation module. The primer sequences are detected in the reference sequence and the reference amplicons are extracted from the reference sequence. The reference amplicons, primer information, and genomic reference information are stored in the references database.

### Mapping the sequenced amplicons

Recently, several fast and elegant mapping algorithms have been developed such as Bowtie, BWA or SOAP [14]. Unfortunately, these algorithms were developed for short-read mapping, typically SOLiD or Illumina reads, and are thus not suitable for mapping of 454/Roche GS-FLX reads. In the VIP pipeline, sequenced amplicon sequences (reads) stored in the raw sequences database are mapped on the reference amplicons using BLAT. The output is parsed using Perl, the Bio::Tools::BPlite package and some custom-made Perl modules (included in the pipeline package).

In amplicon resequencing experiments spanning large exons, overlapping amplicons are used to cover the exon completely. Using the amplicon as a reference template speeds up the mapping process but can cause sequenced reads to map on multiple, partially overlapping amplicons. This problem is addressed by using the mapping position on both the reference sequence and the read. The read should map completely at the beginning of a reference amplicon to be considered as a correctly mapped read.

Sequences with a mapped region (including the PCR primer) shorter than a certain threshold value (default value is 40 bp) are considered to be too short to be a real amplicon. The pipeline will actively look for another mapped region (also shorter than the threshold value) in the sequenced read. When two such regions at both the start and end of a read are detected, both are stored in the database flagged as 'short'.

Users who might prefer to map reads genome-wide - to detect PCR artefacts for example - have the possibility to map on the entire genome, rather than on reference amplicons, using the genome-wide reference database (which is included in the package). Further downstream processing is the same for both approaches.

### Detecting variation in the mapped data

Once the sequenced reads are mapped on the reference template and the best mapping position is determined, the possible differences between the sequenced read and the reference template along with their position relative to the reference template are determined. The VIP pipeline is capable of detecting deletions, insertions and combinations of deletions and insertions.

Rather than storing the alignment itself in a database, the reference amplicon name and the variations are stored in the database. A small sequence window around the variation together with the quality score on the position of the variation is stored in the database as this data is used in later stages of the pipeline.

### Reporting

Once the processed data is stored in the database, the VIP pipeline generates eight standard reports that give end-users ample information to interpret the raw data (Additional file 1). Each of these reports can be generated independently from each other, comes straight from the database and is completely independent from the raw sequence files or raw data processing. Hence, any computer that is connected to the database server can generate its own reports in a short period of time without having access to the raw data.

Reports are mainly generated using SQL-statements and the report generating scripts are partially parallelized using the Perl packages threads and threads::shared. The report generating scripts are parallelized where possible, but one should bear in mind that hard disk accessibility and memory usage are a bigger issue than processing power when using SQL-statements (Additional file 2).

### Estimating the error profile

Detecting variants followed by interpretation is the most important goal of amplicon resequencing projects. The mapping step detects variation by using BLAT and stores the raw variants in the database. These include real variants but also sequencing errors. When reporting to the end-user the VIP pipeline is able to discriminate between sequencing errors and real variants and is optimized to reduce false negatives which is of crucial importance in a diagnostic setting.

In theory, genomic variation is homozygous or heterozygous, thus the frequency of a certain observed variant in an amplicon has to be 50% or 100% of the total reads. In practice, this variant frequency can vary due to sampling variation, sequencing errors, and biological heterogeneity. At low coverage, variants can deviate from the 50% or 100% frequency and make discrimination between homo- and heterozygous variants difficult.

It is known that much of the sequencing errors occur when the basecaller software has to determine the exact length of a stretch of the same nucleotides (homopolymer sequences) [15]. Homopolymer error rates were calculated by looking for homopolymer stretches and counting homopolymer associated deletions and insertions. Although the overall GS-FLX error rate is reported to be relative low (≈0.035%; data not shown), the homopolymer related error rate is at a considerable level (≈10% for homopolymers with length 6 bp and up to 50% for homopolymers with length 8 bp; data not shown) and causing difficulties when calling certain variations as a true variant or a sequencing error [16]. The art of variation analysis is to pick the true variants from the total pool of observed variants. Taking all this into account, a filter was designed that automatically separates sequencing errors from real variants.

### Alignment visualizer

Sometimes, the best way to determine which variants are real and which are not is by looking directly to how the reads were aligned to the reference sequence. The VIP pipeline contains an alignment visualizer that gives a global overview of how several reads were aligned to a reference sequence. The visualizer outputs results per MID/amplicon combination which users can specify. An example output of the visualizer is given in additional file 3.

### The VIP validator

The VIP validator verifies if a certain introduced variant can be detected by the pipeline in a background of existing variants and sequencing errors. Variation is introduced in sequenced reads rather than in the reference template to simulate analysis of real data.

The validator combines the raw sequences database and the mapped sequences database to retrieve a set of raw sequences that map on a certain amplicon which eventually leads to a set of raw sequences associated with the specific amplicon. These reads already contain sequencing errors and real variants. The validator introduces a new variation (SNV, deletion, insertion or combination) at a certain position in a certain amount of sequences in this set. This variation can be something random (i.e. a random variant at a random position) or a predefined variant.

These variations are introduced with a certain frequency (from 0% to 100%) and with a certain coverage by introducing it in a random subset of sequences that are present in the total set of sequences. This way a dataset is generated as if it has been sequenced.

This newly generated dataset is then fed to the mapping and variation detection module of the normal analysis pipeline and the results are further processed in a validation reporting step (Figure 1).

## Results & discussion

The pipeline is designed to process (multiplexed) amplicon resequencing experiments, a setup which is becoming frequently present in diagnostic labs (to replace classic Sanger sequencing) where several genes from several patients are tested together in one sequencing run.

The modular approach makes efficient planning possible. Time-consuming reporting steps can for example be postponed to a time when there is sufficient server power available. This is especially interesting when multiple runs need to be analyzed in a short period of time and efficient server usage is required.

The database approach makes it possible to easily generate reports and draw conclusions from a subset of sequences of a sequencing run or multiple runs together (Additional file 4). It also prevents the user from losing data as all the data is centralized in a single location.

### Amplicon pools

The performance of the pipeline was initially assessed by analyzing two BRCA1/2 resequencing experiments (De Leeneer et al., in preparation). These two runs contained samples that had been analyzed before using classic HRM (high resolution melting) and consequently the variants in the samples were known. Both runs had a similar experimental design.

A total of 111 amplicons (44 BRCA1, 67 BRCA2) were equimolarly pooled together per sample (patient). PCR products of 11 patients (tagged with MIDs) were then equimolarly pooled together. Amplicon sizes ranged from 136 bp to 435 bp (mean 244 bp). The first run generated 542,532 reads (mean length 244 bp); the second run generated 261,646 reads (mean length 247 bp).

### Processing the raw data

During the trimming step (processing the raw data), 97.37% of the raw sequences were split into MID sequence, linker sequence, amplicon sequence and trailing sequence. A small fraction of sequences had a start with too many sequencing errors to determine the correct MID, and these sequences were consequently not used in further analyses (but are nevertheless stored in the raw sequences database). At the moment there is no straightforward method to split sequences into MID, linker and amplicon sequence using the AVA software (AVA only allows MIDs to be split off), which makes it difficult to compare the splitting algorithms, but with a 97.37% yield one can assume that few improvements can be made.

### Mapping the reads

BLAT mapped 85.24% of the sequenced reads generated in the first run and 93.57% of the reads generated in the second run. Some of the mapped sequences were filtered out because they map to a reference amplicon in two pieces with big gap in between and appear to be clear primer-dimers (the so-called 'short' sequences). 76% of all the sequences reads mapped correctly and passed filters in the first run compared to 92% in the second run. The residual portion of the sequenced reads that did not map were further investigated and appeared to be PCR artefacts such as complex primer dimers. Only 4,653 reads (completely or partially) mapped outside the target regions when mapped genome-wide and only 45 of these mapped completely (i.e. from start to stop) somewhere in the genome, from which we concluded that the PCR reactions were specific. Coverage per amplicon and per MID was heterogeneous and between 0 and 5,134 (mean 310) for run 1, and between 0 and 3,019 (mean 175) for run 2. This heterogeneity is mainly caused by differences in PCR efficiency and suboptimal pooling of samples. This heterogeneity (or 'spread factor') can be lowered by carrying out improved pooling strategies and incorporating normalization steps (Hellemans et al., in preparation). Errors in the labwork and/or PCR reactions not working as expected caused some amplicons to be not covered (2.98% in the first run, 1.51% in the second run).

Mapping on reference amplicons rather than on a reference genome certainly improves speed, but is somewhat discussable because one might miss paralogous amplification products. However, in a diagnostic resequencing setup one is interested in variants in a certain set of genes or even a certain exon using thoroughly validated PCR reactions. Paralogous amplification products might become apparent when mapping genome-wide (and be absent when mapping only to reference amplicons) but they would lack diagnostic significance as they give no or incorrect information on the region that was intended to be screened. Users should strive to the use of validated PCR reactions and omitting genome-wide mapping, rather than mapping genome-wide. Nevertheless, the genome-wide approach is included in the package for users desiring to screen for aspecific PCR products.

### Calling true variants

The two BRCA1/2 runs contained samples with known variants (132 distinct sequence variants of which 90 were deletion/insertion mutations).

By setting the filters (using the 'generate reports' script) at the default values one can discriminate between true variants and sequencing errors. The recommended values are: variation frequency (>33% and <67%; >0.95%) and coverage (>20). However, the coverage filter and frequency filter is dynamic and can be changed and exact filter values can be calculated in function of the desired detection power (Hellemans et al., in preparation).

**Figure 2:**
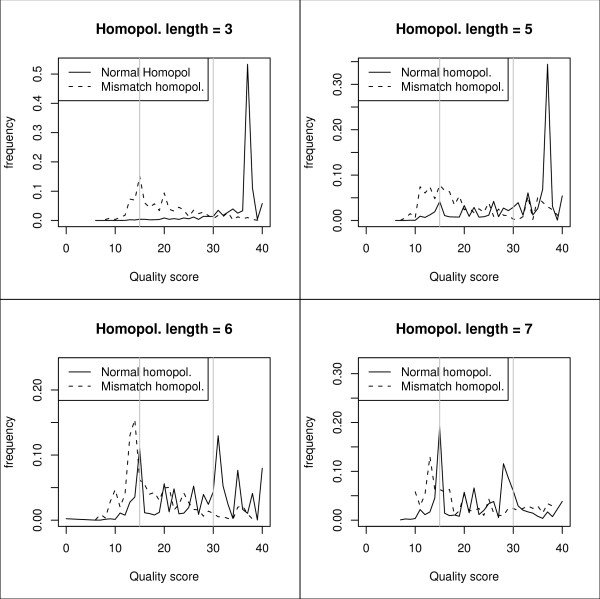
**Influence of quality score (Q) on homopolymer accuracy**. Distribution of homopolymer related quality scores (Q score). The normal homopolymer Q score distribution is determined by making a distribution of the Q score of the homopolymer base; the mismatch homopolymer Q score distribution is determined by making a distribution of the Q score of the base preceding a homopolymer related deletion or the Q score of a homopolymer related inserted base. Distributions are shown for homopolymers with length 3, 5, 6 and 7 bp. The grey vertical lines are drawn at a Q score of 15 and 30. Distributions are based on data from the two BRCA runs.

It is difficult to reliably design a filter to only filter out faulty homopolymer variants. Discarding every variation preceding or following a homopolymeric region is not an option as real variants can also flank such regions. We have seen that for homopolymers < 6 bp there is no problem discriminating between correctly sequenced homopolymeric stretches and homopolymer related insertions or deletions (sequencing errors) by using the quality score (Q). When using the recommended filter value Q > 30 only a minor fraction of the homopolymer related sequencing errors pass the filter (homopolymers < 6 bp). When the stretches are 6 bp long or longer the distributions of the normal and the mismatch homopolymers start to overlap and even a correctly sequenced base has a low Q score (figure 2). We recommend to set the homopolymer filter at 6 bp and keep in mind that no real variants preceding or following a 6 bp homopolymeric stretch can reliably be detected using the default filter values.

In total 97% of all known variants (homozygous and heterozygous) could be detected (sensitivity). Specificity (the portion of called variants that actually are real variants) was 98.5% which means that the false positive rate is only 1.5%. All non-detected variants were insertions/deletions in or near homopolymeric regions. We are aware of the fact that the given numbers may be overfitted to a BRCA1/2 screening, but very similar results were obtained in other experiments (20 genes, 4 runs) as well (data not shown).

**Figure 3:**
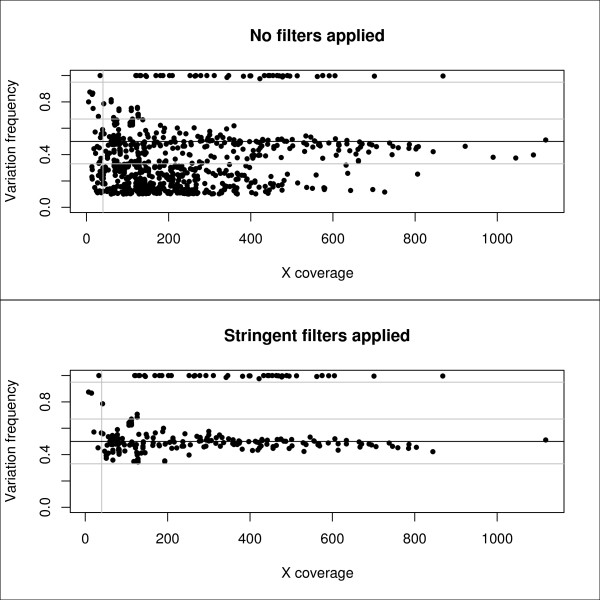
**Pre and post filtering variants data**. Plot of the coverage (times a single sequence is read by the sequencing equipment) and the frequency of an observed variation. In reality, genomic variation occurs at a frequency of either 50% or 100% of total reads. The top figure is the distribution when no filters are applied to discriminate between sequencing errors and real variants; the bottom figure is the distribution where several filters (frequency filter, Q score filter, coverage filter and homopolymer filter) are applied to discriminate the real variations from the sequencing errors. Vertical grey line indicates 40 × coverage; horizontal grey lines indicate respectively 33%, 67% and 95% variation frequency.

A comparison of the data pre- and post-filtering is given in figure 3. The figure clearly shows that the pre-filter data contains a lot of 'noise'. Post-filter data is concentrated around the 50% and 100% level as expected and allows easy discrimination between heterozygous and homozygous variants. At higher coverage, the data is concentrated in a band which is narrower than the specified filter settings. This indicates that at higher coverage, mainly low Q-score variants and homopolymer-related variants are filtered out.

### VIP compared with AVA

The performance of the VIP pipeline was compared with the performance of the AVA software (2.0.0.0). Reads from 1 patient (MID1) containing 67 different amplicons were analyzed in detail. The sample was known to contain 12 variants.

The AVA software does not handle the linker sequence between the MID adaptor and the PCR primer very well. The sequence of the MID/Linker combinations (MID1' = MID1/linker, MID2' = MID2/linker etc.) were used as new MIDs in AVA to circumvent the inability of AVA to split off the linker sequences. The AVA analysis was carried out as described in the manufacturer's manual.

The VIP pipeline returned 50 variants compared to the 235 returned by AVA. This is explained by the fact that the VIP pipeline has an internal filter which filters out any variant with a frequency lower than 10% and with a coverage of only 1. These variants are considered random sequence errors.

Setting a minimum frequency filter in AVA to 20%, 33 variants were identified, whereas 14 variants passed the different filters in the VIP pipeline. The VIP pipeline picked up all 12 known variants (including 6 difficult homopolymer related variants) and called only two false positives, both homopolymer related. The AVA software on the other hand missed all 6 homopolymer related variants and called 27 false positives (Table 1). A detailed overview of all the variants detected by both AVA and VIP is given in additional file 5.

**Table 1:** Comparison of AVA and VIP performance (1 sample, 67 amplicons, 12 known variants)

	AVA software	VIP pipeline
Total variants (unfiltered)	235	50
'Pass filter' variants	33	14
True variants called	6/12 (50%)	12/12 (100%)
False positives	27/33 (81.2%)	2/14 (14.2%)
False negatives	6/12 (50%)	0/12 (0%)

### Additional reports

Besides generating an overview of the variants, the pipeline can also generate additional reports which make the pipeline very useful in a diagnostic setting. All the data is intelligently stored in a relational database and therefore custom analyses can be carried out using SQL-statements in either an SQL browser such as HeidiSQL [17] or by writing custom scripts in any scripting or programming language that has the ability to communicate with a MySQL database.

**Figure 4:**
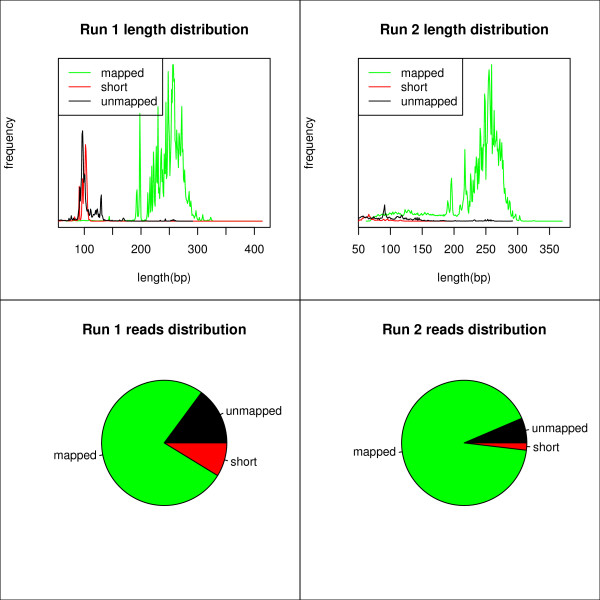
**Improving future sequencing efficiency using priors sequencing data**. Example of the reporting possibilities. Run 1 had many unmappable and short, mapped sequences. Length distribution showed these were mainly 60-120 bp sequences. In Run 2 optimized PCR reactions and an additional length separation were carried out prior to the sequencing with a huge reduction (8% vs. 24%) of unmapped and short sequences and thus improving the cost-effectiveness.

This database approach allowed optimization of the laboratory work of the BRCA1/2 sequencing experiments, especially the pre-sequencing PCR reactions. Using the data from the database, it was possible to detect which amplicons were underrepresented. It was also clear that there were a lot of short sequences in the first run. Run 1 had 8.73% of total reads flagged as 'short sequence', run 2 had 1.87% of total reads flagged as such. Using this information, the multiplex reactions were optimized and an additional length separation was carried out. These actions improved the efficiency by reducing undesired by-products and/or primer dimers from 24% to 8% of the total sequences (Figure 4). It is vital to reduce by-products such as dimers in the early PCR steps as these shorter sequences get preferentially amplified in the emulsion PCR and reduce efficiency even more.

There are 8 standard reports integrated in the reporting module. Each of the reports is described in detail in the manual and an overview is present in the additional files.

### Validating variation using the VIP Validator

Random variation in the VIP Validator is defined as a random error that is introduced with a certain frequency at a certain position in a set of sequences that map on a random reference amplicon. The Validator introduces the variant into a number of sequences and then verifies in how many of these sequences this specific variant was detected at the exact location where it was introduced.

Random variation validation was initially used to optimize and validate the VIP analysis pipeline but has proven to be an effective instrument to detect problematic amplicons or problematic regions in certain amplicons. There are amplicons for which few variants have been reported yet, meaning that amplicon resequencing experiments can unravel new variants. A simulation with random variations can give a clue about regions where accurate variation detection is difficult; for example multiple variations close to each other or variations in repetitive regions can be problematic for the mapping software. The Validator does not explain why a certain region becomes a difficult region to detect variants but informs about variants that cannot be detected using the pipeline.

Random variation was introduced by choosing a random amplicon, a random position, a random variant, a random frequency, and a random coverage and then introducing the variant accordingly. This process was repeated 1000 times.

The ratio between the number of sequences in which a variation was introduced (the coverage so to speak) and the number of sequences wherein the variation was detected is the detection ratio. This ratio is independent from the frequency by which a variant was introduced.

It is clear that SNV detection with the VIP is no problem as 100% of the introduced variants can be detected by the VIP Validator with a sufficiently high detection ratio. The 67% threshold is considered as sufficiently high because the detection ratio for a heterozygous variant would be at least 33.5% and still pass the 33% filter.

**Figure 5:**
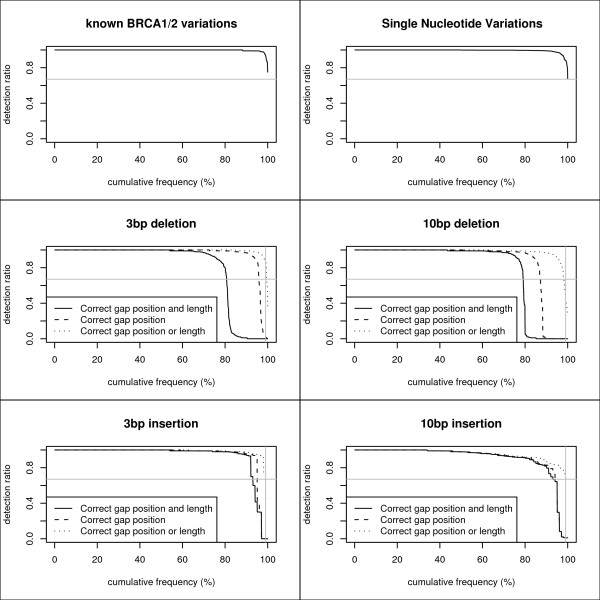
**The VIP Validator**. Detection ratios of 1000 known BRCA1/2 variants, random SNVs, random 3 bp and 10 bp deletions, and random 3 bp and 10 bp insertions. The grey horizontal lines indicate 67% detection ratio. The grey vertical lines indicate a 99% cumulative frequency.

Deletions (and insertions) are often not detected using the newest NGS mapping and variation detection packages (Additional file 5). The VIP pipeline can detect >99% of random 3 bp deletions with a detection ratio ≥ 67%. Determining the location of a gap appears to be relatively easy. The difficult part of gap detection is determining the exact length. Results are similar for longer (10 bp) deletions and insertions (both 3 bp and 10 bp). An overview of the detection ratios is given in figure 5.

These observations make it clear that deletions and insertions can be detected but one should keep in mind that the exact length of the insertion/deletion that passes through the filters is not necessarily the real length. This is one of the drawbacks of needing multiple reads to have a reliable call for a single nucleotide position. Nevertheless, it is detected that something is wrong, which is essential in a diagnostic setting. The alignment visualizer can be a useful resource to manually assess the exact deletion/insertion size.

Rather than using random variation, the VIP Validator can also use a list of known variations as input. Validating known variation is the most useful application of the VIP Validator in a diagnostic setting. It gives an answer to the question "If variation × is present in an amplicon, would it then be possible to detect it using the VIP pipeline?". The answer to this question is objectively addressed using the detection ratio parameter.

Around 95% of the known BRCA1/2 variants were detected by the Validator with a detection ratio of 100% meaning that in every single read (wherein the variant was introduced) the variant was detected at the correct position. The other 5% of the variants were detected with a detection frequency that is lower than 100% but still > 67% (Figure 5).

The VIP Validator can also be used to determine minimal needed coverage in silico. The number of sequences wherein variation is introduced can be altered and one can find a minimal coverage that is needed to have a sufficiently high and reproducible detection frequency.

This Validator is useful for pipeline optimization and determination of the ideal cut-off values. It allows the end-user to fine-tune the pipeline to its own needs and to objectively validate the results of a given pipeline modification. Moreover, it allows validation of the analysis software with respect to the detection of certain variation screening, which is very important in diagnostics, and it determines the detection limits of the pipeline, prior to starting a diagnostic screening.

## Conclusions

We have developed an open-source pipeline that is of great interest for diagnostic resequencing projects. The AVA software (the standard GS-FLX software package) is user-friendly and useful in a research setting but not exactly what is needed in a diagnostic setting. The VIP pipeline is a fully automated pipeline that enables accurate variant detection, even insertions and deletions, and can objectively quantify the identification power by using the Validator. Although the pipeline performs better than AVA when detecting homopolymer related variants, one should bear in mind that reliably detecting variants next to homopolymers will always be an issue, even with high coverage and 'intelligent' software packages. The VIP pipeline also allows a degree of flexibility when one wants to fine-tune its performance, a feature which is lacking in AVA.

The pipeline also allows optimization of the sample preparation procedures and amplicon generating PCR reactions and offers a complete suite of reports that allows researchers in diagnostic labs to assess the reliability of a sequencing run and the detected variants.

The VIP pipeline is under continuous development and improvement as more and more sequencing data becomes available to validate and improve the pipeline. Furthermore, an additional module is in development to do an automated and integrated interpretation of the variants that are discovered using both the amplicon and the genome-wide pipeline.

A package file containing version 1.3 of the pipeline is added as additional file 6.

## Availability and requirements

• **Project name**: Variant Identification Pipeline

• **Project home page**: http://athos.ugent.be/VIP_pipeline

• **Operating system(s)**: Platform independent

• **Programming language**: Perl

• **Other requirements**: BLAT, MySQL

• **License**: GNU LGPL

• **Any restrictions to use by non-academics**: none

## Abbreviations

NGS: next-generation sequencing

## Authors' contributions

JDS wrote the Perl scripts and wrote the manuscript. KDL carried out the sequencing runs and helped estimating the error profile. SL helped estimating the error profile. NS designed a mathematical approach to determine optimal filter values. FP participated in the design of the pipeline. FVN participated in the design of the pipeline. PC, DD, JV, SB, JH, WVC provided access to the sequencing facility and were involved in the design of the pipeline from the very beginning. All authors read and approved the final manuscript.

## Supplementary Material

Additional file 1 **Overview of the standard reports that can be generated using the pipeline**. Overview of the standard reports that can be generated and the time needed to generate the files. Click here for file

Additional file 2 **Report generation time when using multi-threading**. Clock time needed to perform a reporting action using SQL statements and the effect of using multiple threads. Increasing threads reduces the clock time significantly in the beginning, but using more than 7 threads (and thus 7 cores in the server) has no beneficial effect. Averages and standard deviation shown for 6 repetitions. Click here for file

Additional file 3 **Example output of the alignment visualizer**. Screendump of the output generated by the alignment visualizer. Some annotation is added to the figure to explain the format of the output. Bases between 56 and 224 are omitted to ease displaying. Click here for file

Additional file 4 **Doing meta-analyses**. An example of the possibilities of meta-analyses. Three different runs were grouped together in an analysis of the average Q score in function of the read position. Data of more than 1 million reads was used in the analysis. Click here for file

Additional file 5 **AVA compared with the VIP pipeline**. An overview of the variants detected by both the AVA software and the VIP pipeline. The file shows variants that are detected by both AVA and VIP and variants detected by only AVA or VIP. The file also shows detection frequencies and other parameters calculated by both AVA and VIP. Click here for file

Additional file 6 **The VIP pipeline package**. A RAR-file containing the pipeline package (version 1.3), the manual and example data. Click here for file
