# Supplementary material for: Analysing 454 amplicon resequencing experiments using the modular and database oriented Variant Identification Pipeline
Source: BMC Bioinformatics. 2010 May 20;11:269. doi: 10.1186/1471-2105-11-269 (PMC2880033; doi:10.1186/1471-2105-11-269)
Supplement: Additional file 1 — Overview of the standard reports that can be generated using the pipeline. Overview of the standard reports that can be generated and the time needed to generate the files. [file 1471-2105-11-269-S1.DOC]

## Additional file 1: Overview of the standard reports that can be generated and the duration

| Report | Dur. | Paral. | Thr. dur. |
| --- | --- | --- | --- |
| Coverage analysis per MID | 23 sec. | no | 23 sec. |
| Length distribution | 24 sec. | no | 24 sec. |
| Short sequences analysis | 56 sec. | no | 56 sec. |
| Coverage analysis per amplicon | 94 sec. | no | 94 sec. |
| Quality score analysis | 02 min. | no | 02 min. |
| Coverage analysis per MID/amplicon | 18 min. | yes | 04 min. |
| Variation analysis | 07 h. | yes | 02 h. |
| Coverage analysis per single base | 41 min. | no | 41 min. |
| Total time needed for all reports | ± 8 h. | yes | ± 03h. |

Dur.: Time needed to generate the reports of a single GS-FLX run (300,000 reads); Paral.: Is the report generation parallelized? Thr. Dur.: Time needed to generate the report using multiple threads (7 threads).
