# Supplementary figures and images for: Analysing 454 amplicon resequencing experiments using the modular and database oriented Variant Identification Pipeline
Source: BMC Bioinformatics. 2010 May 20;11:269. doi: 10.1186/1471-2105-11-269 (PMC2880033; doi:10.1186/1471-2105-11-269)

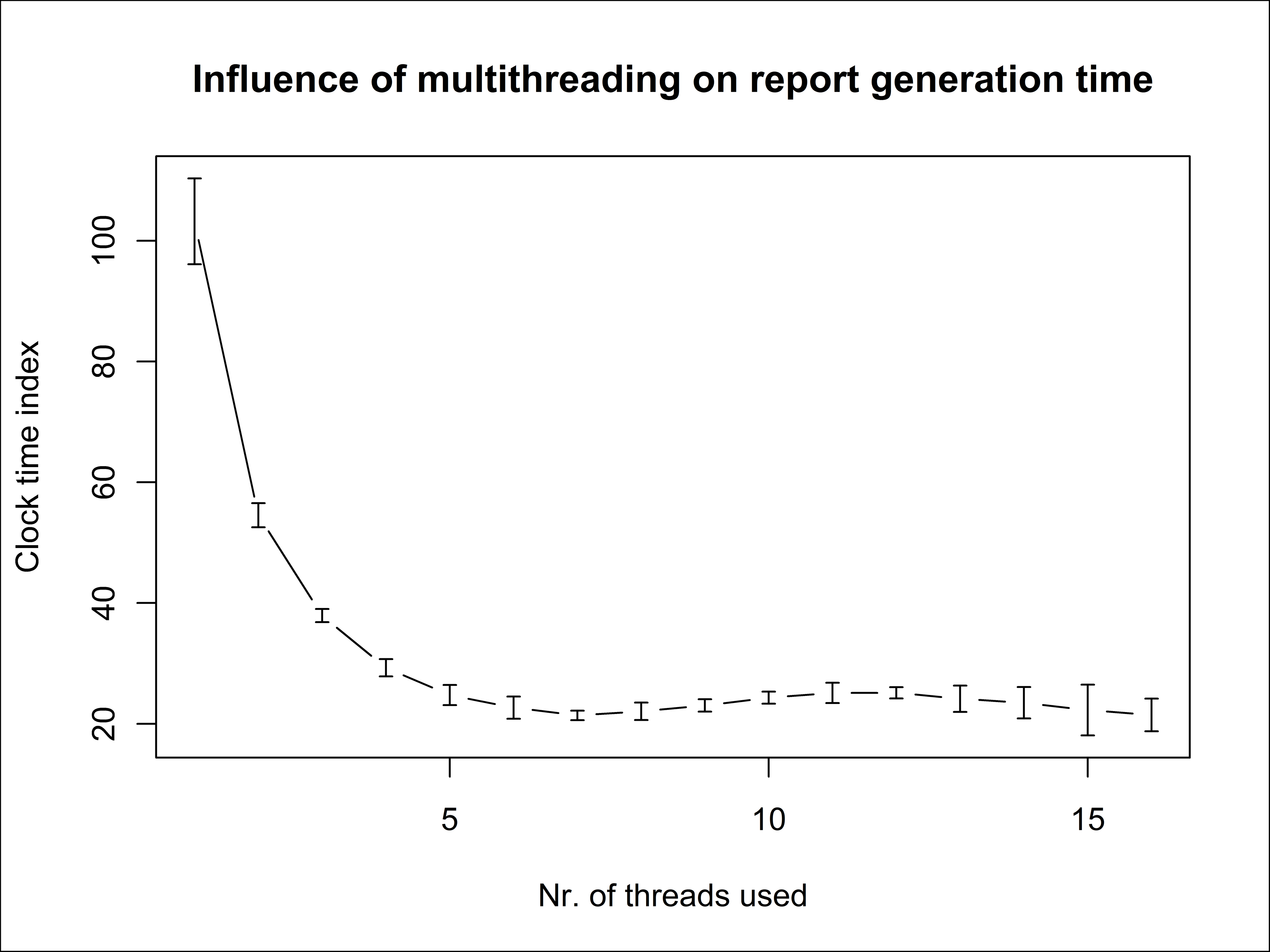

Supplement: Additional file 2 — Report generation time when using multi-threading. Clock time needed to perform a reporting action using SQL statements and the effect of using multiple threads. Increasing threads reduces the clock time significantly in the beginning, but using more than 7 threads (and thus 7 cores in the server) has no beneficial effect. Averages and standard deviation shown for 6 repetitions. [file 1471-2105-11-269-S2.TIFF]

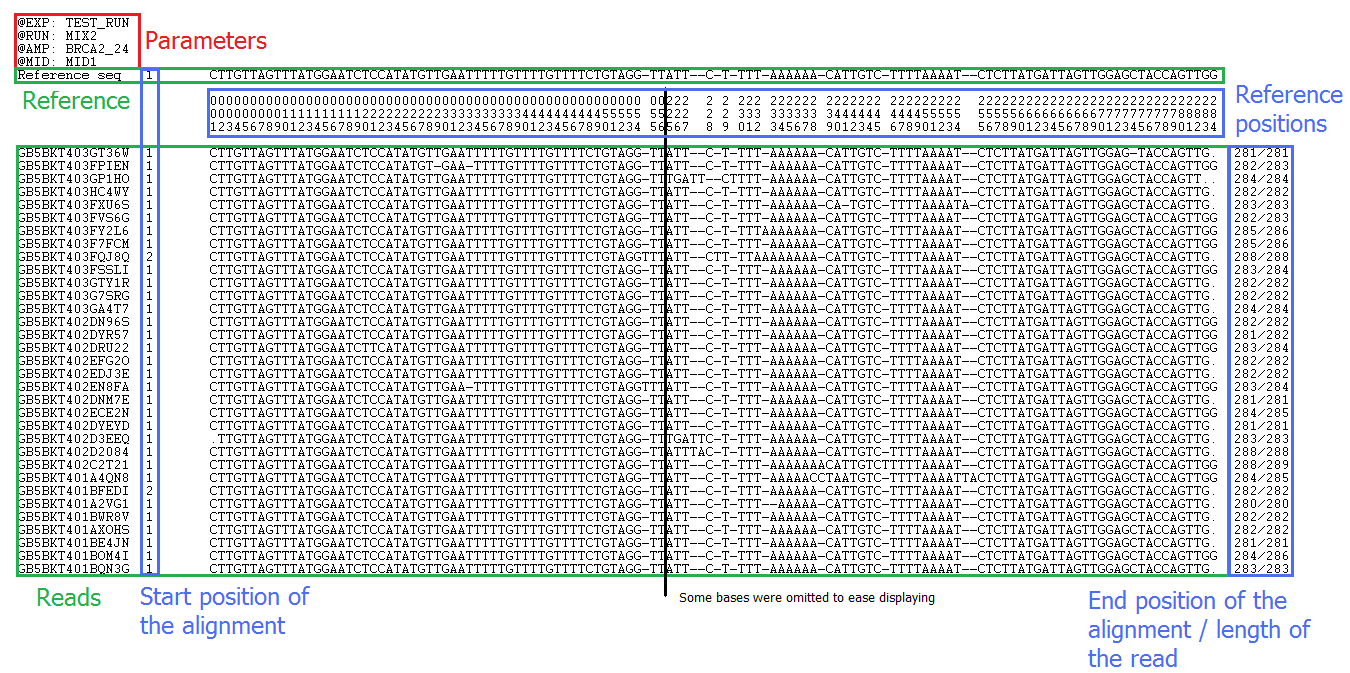

Supplement: Additional file 3 — Example output of the alignment visualizer. Screendump of the output generated by the alignment visualizer. Some annotation is added to the figure to explain the format of the output. Bases between 56 and 224 are omitted to ease displaying. [file 1471-2105-11-269-S3.PNG]
