# Supplementary material for: Analysing 454 amplicon resequencing experiments using the modular and database oriented Variant Identification Pipeline
Source: BMC Bioinformatics. 2010 May 20;11:269. doi: 10.1186/1471-2105-11-269 (PMC2880033; doi:10.1186/1471-2105-11-269)
Supplement: Additional file 4 — Doing meta-analyses. An example of the possibilities of meta-analyses. Three different runs were grouped together in an analysis of the average Q score in function of the read position. Data of more than 1 million reads was used in the analysis. [file 1471-2105-11-269-S4.DOC]

## Additional file 4: Meta-analysis of the quality scores with data from 3 sequencing runs grouped together.


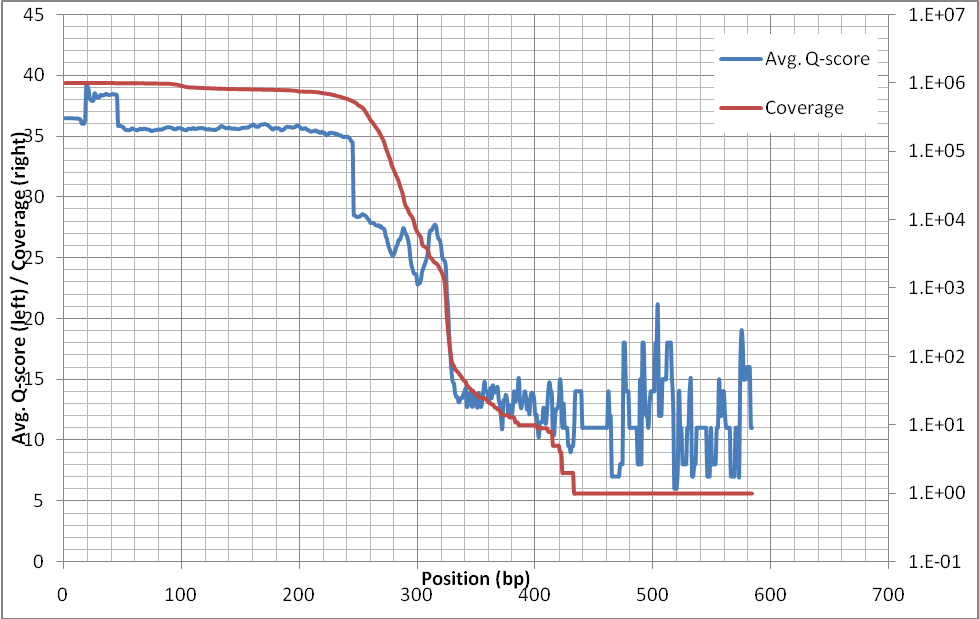


The averages Q-score is shown in blue (left y-axis), the coverage is shown in red (right y-axis). The x-axis shows the position on a single read. Data displayed is coming from more than 1,000,000 reads that originate from 3 different runs.

These analyses can be carried out by modifying the existing reporting modules slightly. The SQL queries can be adapted to narrow down the analysis range (by for example specifying a certain lane) or to expand the analysis range (by for example omitting the ‘WHERE Run=’ part of a query. (Several meta-analysis scripts are available upon request).
